# Editorial for the Special Issue: “Advanced Nanomaterials for Electrochemical Energy Conversion and Storage”

**DOI:** 10.3390/nano12203579

**Published:** 2022-10-12

**Authors:** Zhihong Zhang, Rongming Wang

**Affiliations:** Beijing Advanced Innovation Center for Materials Genome Engineering, Beijing Key Laboratory for Magneto-Photoelectrical Composite and Interface Science, Institute for Multidisciplinary Innovation, School of Mathematics and Physics, University of Science and Technology Beijing, Beijing 100083, China

Developing efficient and low-cost energy conversion and storage devices and technologies is all-important issue in order to achieve a low-carbon society, whose performance essentially depends on the properties of materials. Nanomaterials have been extensively demonstrated to have great potential applications in energy conversion and storage devices and technologies, i.e., batteries, capacitors, electrocatalysis, and nanogenerators. On the other hand, to realize their practical use, further optimization of the nanomaterials’ structures and properties is still needed.

This Special Issue aims to communicate the recent advances of advanced nanomaterials for energy conversion and storage. It covers the design, synthesis, properties, and applications of advanced nanomaterials for energy conversion and storage. Twelve research works focus on various nanomaterials for batteries, capacitors, electrocatalysis, nanogenerators, and magnetic nanomaterials. Furthermore, two reviews present two kinds of two-dimensional (2D) materials, namely 2D amorphous nanomaterials and phosphorus-doped graphene in electrochemical energy conversion and storage. A brief overview of the published articles is presented in the following, and we hope to provide useful information for potential readers.

Lithium–sulfur (Li-S) batteries are receiving increasing attention as next-generation high-energy-density storage systems. However, currently, Li-S batteries suffer from low volumetric energy density and poor cycling stability due to the intrinsic low conductivity of sulfur and its discharge product, lithium polysulfide shuttle effect, and so on. Many strategies have been proposed to improve the performance of Li-S batteries, and one effective way is to design the “adsorptive-catalytic” cathode. Chen et al. [1] developed a three-dimensionally ordered macro/mesoporous Nb_2_O_5_/Nb_4_N_5_ through in situ nitridation to serve as a multi-functional sulfur host. The strong adsorption of Nb_2_O_5_ and high conductivity and catalytic activity of Nb_4_N_5_, combined with the porous structure, enable batteries with the S/Nb_2_O_5_/Nb_4_N_5_ cathode to exhibit excellent cycling stability and higher discharge capacity. Wang et al. [2] adopted similar concept to construct composite cathodes. They synthesized TiN@C/S/Ta_2_O_5_ with high sulfur fraction through a simple and low-cost co-precipitation method. Benefiting from the high conductivity of TiN, the strong adsorption of Ta_2_O_5_, and the micro- and mesoporous structure of the multidimensional carbon structure, the batteries with such cathode showed superior cycle stability and high areal capacity with a high sulfur utilization.

Lithium–oxygen (Li-O_2_) batteries also have high theoretical capacity, but in practice their energy density is less than half the theoretical one, due to the great energy loss during the charging and discharging process. Designing highly active catalyst with low cost and great stability is one efficient way to tackle the problem and promote the practical use of Li-O_2_ batteries. Li et al. [3] fabricated a CoFe_2_O_4_/g-C_3_N_4_ composite catalyst with CoFe_2_O_4_ particles supported on the flaky g-C_3_N_4_ using a scalable facile method. Both CoFe_2_O_4_ and g-C_3_N_4_ can provide reactive sites for the discharge–charge reaction, and the flaky g-C_3_N_4_ with a high specific area and high chemical stability enabled a strong mass transfer ability and stable support for restraining the aggregation of CoFe_2_O_4_ particles. Under the synergistic effect of CoFe_2_O_4_ and g-C_3_N_4_, the Li-O_2_ batteries exhibited improved capacity and stability.

Superconductors are another important energy storage devices with advantages of high-power density and long lifetime. One type of superconductor is the pseudo capacitor, where reversible faradic-type redox reactions occur at the electrode surface. Sylla et al. [4] prepared a composite electrode material for pseudo capacitors, where MoO_2_ and Mo_2_C nanostructures were incorporated into a peanut-shell-activated carbon (PAC) network via a one-step pyrolysis route. The composite combined the high specific area of PAC, the pseudocapacitive effect of MoO_2_, and the superior conductivity and stability of the Mo_2_C, thus delivering excellent capacitive performance when used as electrodes in a symmetric supercapacitor.

In the energy conversion devices, the properties of the catalyst determine their performance, and thus developing low-cost, highly active, and stable catalysts is of great significance to realize high-efficiency and high-selectivity energy conversion. Many nanomaterials exhibited various advantages as catalysts due to their high specific area which can offer abundant active sites. Carbon-based doped nanomaterials are some of most widely studied catalysts. Rong et al. [5] developed a self-template-assisted pyrolysis route to prepare a three-dimensional nanoporous carbon structure co-doped with N/P/Fe. The synthesis process is simple, avoiding the extra process of template removal, and the distribution and content of N/P/Fe can be well controlled. The prepared catalysts exhibited superior oxygen reduction reaction catalytic activity and durability compared to the currently advanced Pt/C catalysts.

Two-dimensional materials have also attracted extensive attention in the catalysis field. Graphene has a high specific surface area, high stability, and excellent conductivity, making it a promising candidate for catalyst. However, the pristine graphene is semimetal with no bandgap and shows poor catalytic activity. Heteroatom doping could greatly alter the electronic properties and improve the catalytic activity of graphene. Zhan et al. [6] reviewed recent advances in P-doped graphene electrocatalysts for the oxygen reduction reaction, including the synthesis and performance of the materials and catalytic mechanism. MXenes, as a new member of 2D materials, have also been demonstrated to have great potential in the electrocatalysis process. Luo et al. [7] studied how the Fe atom adsorption on pure or doped Ti_3_C_2_O_2_ affected the catalytic performance of the nitrogen reduction reaction using the density functional theory (DFT)They found the charge transfer of the adsorbed Fe atoms to N_2_ could promote the hydrogenation of N_2_ and thus improve the catalytic performance. Two-dimensional materials supported single-atom catalysts which exhibit unique catalytic performance, represent one of the hot topics in the catalysis field. Zhao et al. [8] designed a single-atom catalyst Au/WSSe by filling the single Au atom at the S vacancy site in the Janus WSSe monolayer. By DFT calculation, they found the strong binding between the single Au atom and the WSSe resulted from the electron transfer and orbital hybridization between Au and W. Moreover, the tensile strain in the support could further improve the electrocatalytic performance in the hydrogen evolution reaction of the Au/WSSe catalyst. In fact, due to their unique structure and excellent properties, 2D materials have been widely investigated in electrochemical fields, not only the electrocatalyst, but also the batteries and supercapacitor. Liu et al. [9] focused on the 2D amorphous nanomaterials and summarized various regulation strategies, including composition and structure design, to enhance the electrochemical performance for the batteries, supercapacitors, and electrocatalysts.

The nanogenerator is a kind of mirco-nano device that can realize energy conversion. Since the first piezoelectric nano-generator (PENG) came onto the scene, the concept, mechanism, and applications of nanogenerators have been widely studied and made great progress. Zhu et al. [10] developed a self-powered sport sensor which was composed of a triboelectric nanogenerator (TENG), a PENG, and a flexible transparent stretchable self-healing hydrogel electrode. The prepared sensor is stretchable, wearable, and transparent, and can be used to monitor human three-dimensional motions.

Magnetic materials provide an alternate source of clean and renewable energy, and also play an irreplaceable role in energy conversion and storage. Researches on the physics and properties of magnetic materials are essential to realize their optimization for energy application. Gao et al. [11] prepared the ErFe_x_Mn_12−x_ series alloy samples with Δx = 0.2 using the arc melting method, and achieved a detailed magnetic phase diagram of the samples. They also studied the exchange bias effect and magnetocaloric effect in such a magnetic alloy. Nano-magnetic materials with small size and unique magnetic properties have played vital roles in many fields and their properties can be modulated by distinct strategies. Du et al. [12] developed a self-assembly method to prepare a single layer of aligned Co nanorods to obtain improved magnetic performance. They also studied the magnetic interaction of Co nanorods with different shapes, offering guidance to the magnetostatic interaction of shape anisotropic magnetic nanostructures. Gao et al. [13] investigated the magnetic and electronic properties of Janus MXene (M’MCO_2_, M’ and M = V, Cr, and Mn) via first-principles calculations and they found that the transition metal and configuration could tune the band gap, the magnetic ground state, and the net output magnetic moments of the Janus MXene materials. The work points out a new path to design and regulate novel magnetic materials. Cui et al. [14] synthesized laterally asymmetric heavy metal (HM)/ferromagnetic metal (FM) multilayers by growing the FM layer in a wedge shape, and studied field-free spin–orbit torques (SOTs) switching in the asymmetric multilayers. They found that the switching efficiency strongly depended on the HM/FM interface and the FM layer thickness. 

In this Special Issue, some original research works and high-quality reviews on the advanced nanomaterials for energy conversion and storage are presented, and we hope that these articles prove informative and instructive for readers. The research in this field is booming and great advances in the development of efficient, clean, and sustainable energy devices and technologies are expected. 
